# Molecular simulations meet personalized medicine: The mechanism of action of CLC-5 antiporter and the origin of Dent's disease

**DOI:** 10.1093/pnasnexus/pgaf353

**Published:** 2025-11-04

**Authors:** Veronica Macaluso, Carles Pérez, Robert Soliva, Yvonne Westermaier, Lucía Díaz, Miłosz Wieczór, Modesto Orozco

**Affiliations:** Institute for Research in Biomedicine (IRB Barcelona), Barcelona Institute of Science and Technology, C/ Baldiri Reixac 10, 08028 Barcelona, Spain; Nostrum Biodiscovery, Av. de Josep Tarradellas 8-10, 08029 Barcelona, Spain; Nostrum Biodiscovery, Av. de Josep Tarradellas 8-10, 08029 Barcelona, Spain; Nostrum Biodiscovery, Av. de Josep Tarradellas 8-10, 08029 Barcelona, Spain; Nostrum Biodiscovery, Av. de Josep Tarradellas 8-10, 08029 Barcelona, Spain; Institute for Research in Biomedicine (IRB Barcelona), Barcelona Institute of Science and Technology, C/ Baldiri Reixac 10, 08028 Barcelona, Spain; Department of Physical Chemistry, Gdansk University of Technology, Gabriela Narutowicza 11/12, 80-222 Gdańsk, Poland; Institute for Research in Biomedicine (IRB Barcelona), Barcelona Institute of Science and Technology, C/ Baldiri Reixac 10, 08028 Barcelona, Spain; Department of Biochemistry and Biomedicine, University of Barcelona, Av. Diagonal 643, 08028 Barcelona, Spain

## Abstract

ClC-5 is a Cl−/H+ antiporter crucial for the homeostasis of the entire organism, and whose functional deficiencies cause pathologies such as Dent's disease, a rare genetic disorder that can have lethal consequences. While the clinical aspects of the pathology are known, its molecular basis is elusive, which hampers the development of potential therapies. We present here a systematic study, where we explore the mechanism of transport of ClC-5, deciphering the choreography of structural changes required for the transport of chloride ions and protons in opposing directions. Once the mechanism is determined, we explore how the 523ΔVal deletion linked to Dent's disease hampers the correct functioning of the transporter, despite having a very minor structural impact. Our study highlights how state-of-the-art simulation methods can shed light on the origin of rare diseases and explore targets for personalized medicine.

Significance Statement The human chloride/proton antiporter hClC-5 regulates endosome acidification during tubular reabsorption in kidneys, allowing vital small biomolecules and ions to be retained in the body. Because of this crucial role for homeostasis, loss of function of ClC-5 severely impairs kidney function, leading to life-threatening consequences in carriers of pathogenic mutations. While current modes of treatment remain focused on symptoms, understanding the molecular details of how hClC-5 achieves ion exchange and how clinical mutations impair this process holds promise for mutation-specific personalized treatments of the future.

## Introduction

Dent's disease (DD) type 1 is an ultra-rare X-linked disorder characterized by low-molecular-weight proteinuria (LMWP), hypercalciuria, nephrocalcinosis, and/or nephrolithiasis, and it frequently progresses to chronic kidney disease (CKD) (1). While DD patients receive treatment to alleviate and prevent symptoms, there are no curative treatments for the disease. Most cases of DD are linked to alterations of the CLCN5 gene (Xp11.22) coding for the ClC-5 protein, a Cl^−^/H^+^ antiporter (2, 3) expressed in kidney and intestine epithelia (1). In the human kidney, ClC-5 is found in proximal tubule cells (PTCs), colocalizing with H^+^/ATPase in early endosomes, where both regulate acidification. To a lesser extent, ClC-5 is also present in the plasma membranes (PM), where it mediates PM chloride currents and/or participates in the endocytosis of low molecular weight proteins (2, 3). Genomic studies have shown hundreds of ClC-5 variants with potential pathological profile, the most frequent being missense (35%), frameshift (31%), nonsense (16%), splicing mutations (10%), and large deletions (4%), while lower-frequency variations (1–2%) include in-frame deletions, complex mutations, Alu insertions, and 5′UTR mutations (4). Functionally, some of them are related to incorrect folding and degradation in the endoplasmic reticulum, while others lead to defective mutants unable to generate chloride currents (2, 3).

ClC-5 is part of the larger family of ClC channels and antiporters responsible for the transport of chloride, or similar anions, across the membrane. ClC channels catalyze the passive transport of the anions along their electrochemical gradients, while antiporters, such as ClC-5, use the concentration gradient of one ion to transport another one in the opposite direction with a fixed stoichiometry of two anions per one proton (1, 5). Among human transporters, only ClC-7 and ClC-6 have been experimentally characterized (6, 7), but comparison of algae and prokaryote transporters and other mammalian channels (EcCLc, StCl, CmCLC, CLC-K, and ClC-1) suggests that despite the different permeation mechanisms, ClC channels and antiporters show large structural similarity (8–12). Thus, all of them are pseudosymmetric homodimers, where each monomer comprises a transmembrane (TM) domain, while in eukaryotes, an additional cytosolic cystathionine beta-synthase (CBS) domain is present. Each TM domain consists of 18 antiparallel α-helices, comprising an ample vestibule at either side of the membrane and a narrow pore, where two glutamic acids act as gates for anion uptake (11, 13).

While the Cl^−^ channel is mostly passive during transport, significant pH-dependent conformational changes are expected for the antiporter (14–17). Thus, a variety of studies have highlighted the coupling of ion transport with the conformational and protonation states of the gating glutamates (Glu_ext_ and Glu_int_ pointing towards the extracellular and intracellular sides, respectively) (15, 18–20), as well as with significant conformational rearrangements in the TM helices affecting the Cl^−^ pathway (15, 17). Past studies in model organisms also demonstrated that small changes away from the main translocation pathway can produce orders-of-magnitude variation in antiport kinetics (21). However, despite all the effort focused on this system (10, 15, 19, 22, 23), the precise sequence of events responsible for ion passage is still unclear, hampering the possibility to define the molecular origin of the pathological nature of certain genomic alterations, like those responsible for DD.

In this contribution, we describe the mechanism of ion transport of ClC-5 and how it is altered by the ClC-5 523ΔVal in-frame deletion, a poorly characterized rare genetic alteration associated with DD (2, 3, 24, 25), for which a knock-in mouse model is available (26). By combining a variety of *state-of-the-art* simulation techniques for the wild type (WT) and 523ΔVal variant, we obtain a detailed model of the 2:1 antiport mechanism, deciphering a series of coordinated movements coupled to the inverse flux of Cl^−^ and H^+^. By comparing the transport mechanism of the wild type (WT) and the 523ΔVal variant, we define the molecular basis of the 523ΔVal pathogenicity and highlight some structural differences that might be used to design drugs that recover the normal functionality of the antiporter.

## Methods

### Structure preparation and molecular dynamics simulations setup

**Fig. 1.**
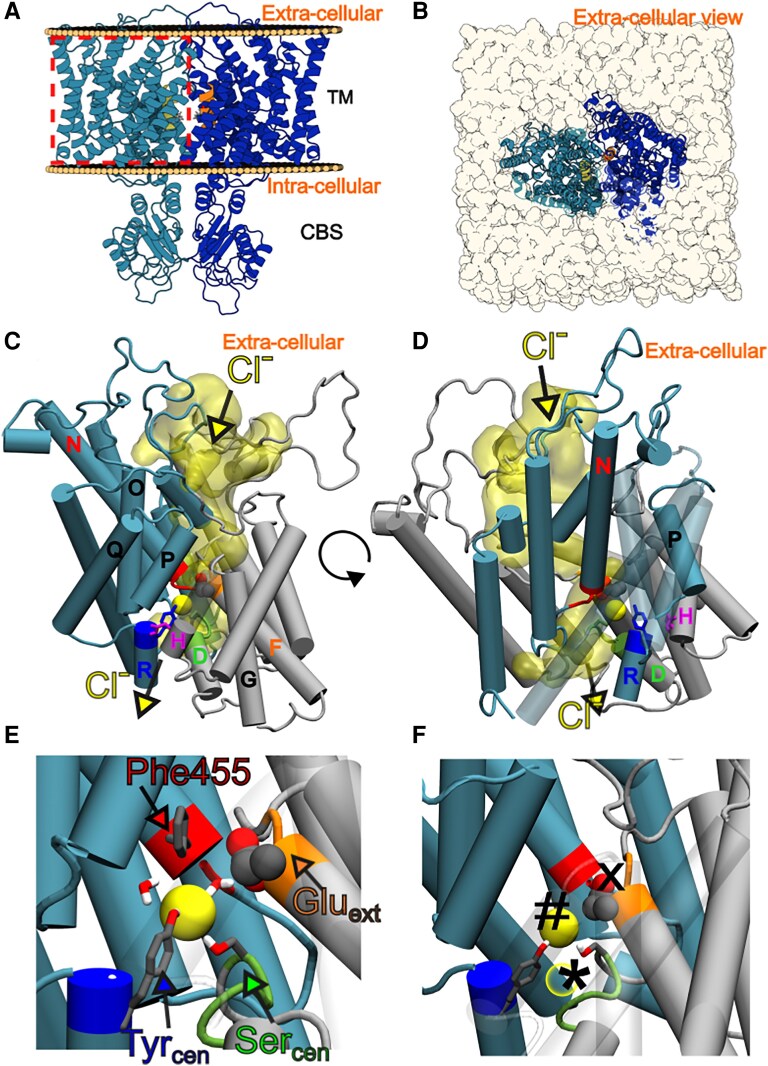
Wild-type ClC-5 homodimer (A, B), details of the TM domain (C, D), and binding sites representation (E, F) in two side views. A and B) Side and top (extracellular) view of wild-type ClC-5, respectively are shown. Chains A (left) and B (right) are in blue and dark blue, with the α-helix P (where Val523 is placed) is yellow (chain A) or orange (chain B). C, D, E, and F) Details of one of the wild-type monomers, with α-helices A to I in gray, and α-helices J to R in cyan are shown. Electropositive N-termini of α-helices D (green), F (orange), and N (red) and key residues for the ClC-5 antiporter mechanism are highlighted (Tyr_cen_, Ser_cen_, and Glu_int_ licorized blue and green and magenta respectively and Glu_ext_ in CPK; global pictures are shown in (C) and (D) and more detailed representation are shown in (E) and (F). Yellow arrows indicate Cl^−^ ions pathway. E and F) A molecular representation of chloride binding sites is shown: S_ext_ (labeled with “X”) is occupied by Glu_ext_ side chain; S_cen_ (labeled with “#”) is occupied by a chloride anion (yellow solid sphere); and S_int_ (labelled with “*”) which can be occupied by another chloride (yellow empty sphere). The detailed placement of Val523 in the structure (helix P) is shown in Fig. S2.

As no structure of human ClC-5 is available, and the only resolved human homologs ClC-7 (7) and voltage-gated ClC-6 (6) have sequence identity of mere 31.5 and 30.4%, respectively, we tried different homology modeling strategies to obtain structures for WT and 523ΔVal forms of the ClC-5 transporter, eventually settling on AlphaFold-Multimer predictions (27–29) as implemented in COSMIC2 (30). Predicted structures show high-confidence scores and the expected fold for an antiporter (see Fig. 1), and upon STAMP alignment (31), the TM part has an average per-residue RMSD with respect to ClC-7 (PDB ID 7JM7) of 1.61 Å (see Table S1 for comparison against other ClC proteins, spanning RMSD values between 0.7 and 2.06 Å). Each chain in the ClC-5 dimer comprises the TM and the cytosolic cystathionine beta-synthase (CBS) domains. Missing hydrogens were added using the H^++^ webserver (32), according to their pKa values. By default, the glutamate residue 211 (Glu_ext_) was protonated in both chains, while glutamate residue 268 (Glu_int_) was considered deprotonated. As discussed later, this would correspond to the starting configuration for chloride transport from the cytoplasm to the intracellular space.

The protein structures yielded by AlphaFold were embedded in a phosphatidylcholine (POPC) bilayer and immersed in a rectangular water box (13 × 13 × 15 nm and 13 × 13 × 14 nm dimensions for WT and 523ΔVal, respectively) using CHARMM-GUI Membrane Builder (33). To favor transport, chloride ions (Cl^−^) were added to reach 1 Molar concentration, while sodium ions (Na^+^) were added as needed to charge-neutralize the system. CHARMM36m (34–36) force field was used to model protein, anions, and lipids, while water was modeled using TIP3P (37, 38). The systems were minimized, thermalized, and equilibrated before performing production runs (see Supplementary Methods for details). Small local distortions found in the AlphaFold model were quickly relaxed during equilibration, yielding very stable structures (see Fig. S1). Equilibrium trajectories of three replicas were performed for 1 μs per system (WT or 523ΔVal) using GROMACS2020 software (39). From thus constructed equilibrium ensemble, four frames were selected as seeds for free energy (metadynamics) calculations based on the progression of chloride ions, and ion concentrations were adjusted to the physiological 0.15 M by removing excess ions (see Supplementary Methods for additional details).

To assess the effect of initial protonation states, four additional 250-ns equilibrium runs were performed at 0.15 M that covered two replicas of each possible combination of protonation states of the catalytic glutamates (Glu_int_^0^/Glu_ext_^0^, Glu_int_^0^/Glu_ext_^−^, Glu_int_^−^/Glu_ext_^0^, and Glu_int_^−^/Glu_ext_^−^) in different chains of the dimer. The ensembles from these simulations were compared against metadynamics runs (Fig. S2) and used to identify allosteric pathways with the Dynamic Network Analysis tool (40).

### Tunnels identification

CAVER (41) was used to identify tunnels in the equilibrated structures of WT and 523ΔVal. Representative snapshots for the tunnels analysis were obtained by a *k-means* clustering analysis using the RMSD of residues in proximity of Glu_ext_, Ser_cen_, and Tyr_cen_ as a descriptor (see Fig. 1). To define “productive cavities,” we imposed a condition that tunnels should connect from the bulk (outer surface of the protein at the intracellular or extracellular space) to the external-gate region.

### Multiple-walker metadynamics and pKa calculations

Multiple-walker well-tempered metadynamics (MWWTMetaD) (42, 43) was used to explore the correlated free-energy surface of two chloride ions confined in the central channel. Eight simulations were run for the WT and 523ΔVal variant dimers, for two different protonation states of the gating glutamates (Glu_ext_ protonated and Glu_int_ deprotonated, and Glu_ext_ deprotonated and Glu_int_ protonated). Starting configurations were selected from the ensembles obtained by unbiased molecular dynamics (MD), where the density of Cl^−^ around the entrance of the anion tunnel was high. This process led to 64 independent walkers (8 walkers × 2 independent chains × 2 protonation states × 2 protein forms), symmetrized and combined to obtain four free energy landscapes of the (permutation-symmetric) chloride pairs, named WP (wild type with the Glu_ext_ protonated and Glu_int_ deprotonated state), WD (wild type with Glu_ext_ deprotonated and Glu_int_ protonated), MP (the 523ΔVal mutant form with the Glu_ext_ protonated and Glu_int_ deprotonated state), and MD (the 523ΔVal mutant form with Glu_ext_ deprotonated and Glu_int_ protonated). In all cases, we used two collective variables, one per ion, defining the Z-displacement (i.e. along membrane normal) of the two chlorides from the center of mass of the central cavity (see Supplementary Methods for details). To accelerate convergence, after ca. 1 μs per walker, we averaged and symmetrized hills for both chains, and restarted the simulations with a 4-fold larger initial hill height for another >500 ns per walker. Symmetrization relied on the experimentally confirmed independence of each chain's activity (44), as well as the low allosteric coupling between chains (see Fig. S3). Run parameters of the associated simulations were identical to those used to obtain the unbiased trajectories.

### Effective pKa calculations

We used classical molecular interaction potential (CMIP) (45) (with a nonlinear Poisson–Boltzmann's electrostatic term; see Supplementary Methods) to compute configuration-dependent pH for the two gating glutamic acids. To this end, metadynamics trajectories were binned according to the Z position of both chloride ions, and the calculation was performed on up to 10 selected frames with the highest statistical weights in each bin (a total of 12,906 structures) according to the metadynamics reweighting scheme (46); the pK_a_s corresponding to individual frames were then averaged. In all cases, CHARMM36 charges and van der Waals parameters were used in CMIP, combined with standard dielectric constant for protein and solvent and physiological ionic strength for the Poisson–Boltzmann part of the calculation. Note that the pK_a_s were only calculated for deprotonation, to avoid spurious clashes when protons are added to the PB calculation; for this reason, the reported values correspond to a “deprotonation potential” and are used as a proxy for true equilibrium pK_a_. Accordingly, high values of pK_a_ reported here correspond to frames where the protonated glutamate coordinates the chloride ion, and an instantaneous deprotonation results in strong repulsion between two negative charges. These conditional values, representative of averages over a macrostate defined by the positions of two chlorides, should not be confused with experimentally measurable pK_a_, corresponding to averages over the entire ensemble (including both protonation states). As a consequence, the main purpose of conditional pK_a_s is to locate glutamate deprotonation in a physically plausible sequence of events in the transport cycle. In addition, as we specifically calculate the propensity to deprotonate, the implied ordering of events is not necessarily the same in the reverse direction, where Glu_ext_ needs to be protonated.

To additionally corroborate the main pK_a_ shift observed in Poisson–Boltzmann-based calculations (where proton transfer from the external glutamate is expected), we performed constant-pH titrations for the two corresponding chloride pair positions, using phbuilder (47) and the Gromacs implementation of the constant-pH algorithm (48). For each pK_a_ estimate, eight windows with different pH values were simulated with 35 ns of sampling per window.

### QM/MM metadynamics for proton transfer

We extracted a sample frame that contained a Grotthuss-like water wire composed of three water molecules, and subjected it to QM/MM equilibration in NAMD (49, 50) coupled to MOPAC (51) (set up with help of QwikMD (52)). The quantum subsystem contained the two glutamate residues and three water molecules, and was represented at the PM6-D3H4X semiempirical level of theory (53). The COLVARS library (54) was used to perform metadynamics along a collective variable defined as the difference between the sum of lengths of O–H bonds that are about to be formed, and the sum of lengths of O–H bonds that are formed in the starting configuration. In addition, distance restraints were introduced to (i) keep all the nonexchangeable protons bound to their respective oxygen atoms and (ii) prevent any dynamic O–H distance from exceeding 2.5 Å. A bias of 0.5 kcal/mol with a spread of 0.3 Å was added every 5 MD steps to drive the transitions, and the final free energy profiles were obtained as an average of nine intermediate profiles integrated in intervals of 2,500 Gaussian kernels.

### Determination of druggable cavities

Preliminary druggability studies were performed using Fpocket (55) on 10 selected structure representatives of highly populated clusters from the unbiased simulation of the 523ΔVal variant. A more exhaustive analysis was done using MDpocket (56), a method that allows us to account for cavity plasticity using unbiased trajectories of both WT and 523ΔVal variants (see Supplementary Methods for details).

## Results and discussion

### AlphaFold model and solution structure

Consistently with known structures of other ClC proteins, the structural model of ClC-5 generated by AlphaFold and refined by MD simulations shows a dimeric protein (Fig. 1A and B), where each TM domain consists of 18 α-helices. An ample vestibule (Fig. 1C and D) is present at both sides of the membrane, and a narrow pore, whose center is defined by the N-terminal regions of α-helices D, F, and N, and an R-helix Tyrosine (Tyr_cen_), which generates a central cavity (S_cen_). This cavity acts as an anion trap, and is separated from the cytoplasmic side by the side chains of D-helix Ser_cen_ and R-helix Tyr_cen_. Moving closer to the extracellular side, another cavity, hereafter named S_ext_, is located between the α-helices N and F, and is able to accommodate either the negatively charged side chain of an F-helix Glutamate residue (Glu_ext_) or a chloride anion. Finally, a third cavity S_int_ is located below S_cen_, flanked by the N-terminus of α-helix D on the one side, and the intracellular aqueous vestibule on the other. This cavity is expected to be important for the recruitment of ions from the intracellular environment, or in the chloride exit pathway in the opposite direction of transport (22) (see Fig. 1 for description of the crucial regions of the anion tunnel). These three cavities are in very close proximity, defining a narrow gating site that controls the flux of ions in the channel.

**Fig. 2.**
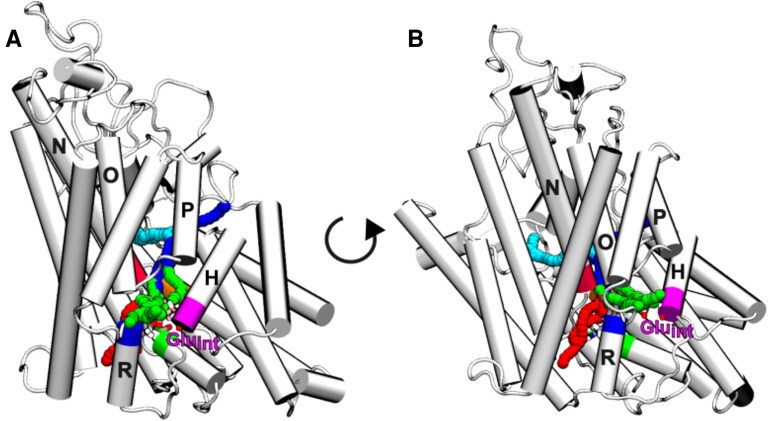
Visualization of the four tunnel clusters obtained for wild type and 523ΔVal systems projected on a representative molecular structure of wild type TM chain. A and B) A different-rotation lateral view of the TM chain is offered. α-helix H, on which Glu_int_ is located, is colored in magenta. The cyan and blue tunnels start from the extracellular vestibule and resemble the extracellular Cl^−^ and H^+^ common path described in the literature for other ClC channels. The red tunnel cluster goes from the intracellular vestibule (figure bottom) and passes in proximity of Tyr_cen_ and Ser_cen,_ representing the intracellular chloride path; the green one starts from the intracellular vestibule near Glu_int_ and represents the intracellular H^+^ path.

According to structures refined through MD, the 523ΔVal deletion leads to minor structural changes: the only noticeable change is a certain compaction between the two subunits (see Fig. S1). The deletion is well accommodated, maintaining the secondary structure on helix P until Arg516, where a short turn recovers the phase of the helix without major changes in the contact residues (see Fig. S4). CAVER analysis (see Methods) shows the existence of four tunnels connecting the extracellular or intracellular vestibule to Glu_ext_, which can be assigned to the preferred H^+^ and Cl^−^ paths (Fig. 2) and which are all present in both WT and 523ΔVal variants.

### Thermodynamics of the mechanism of Cl^−^ transport

#### The chloride migration

**Fig. 3.**
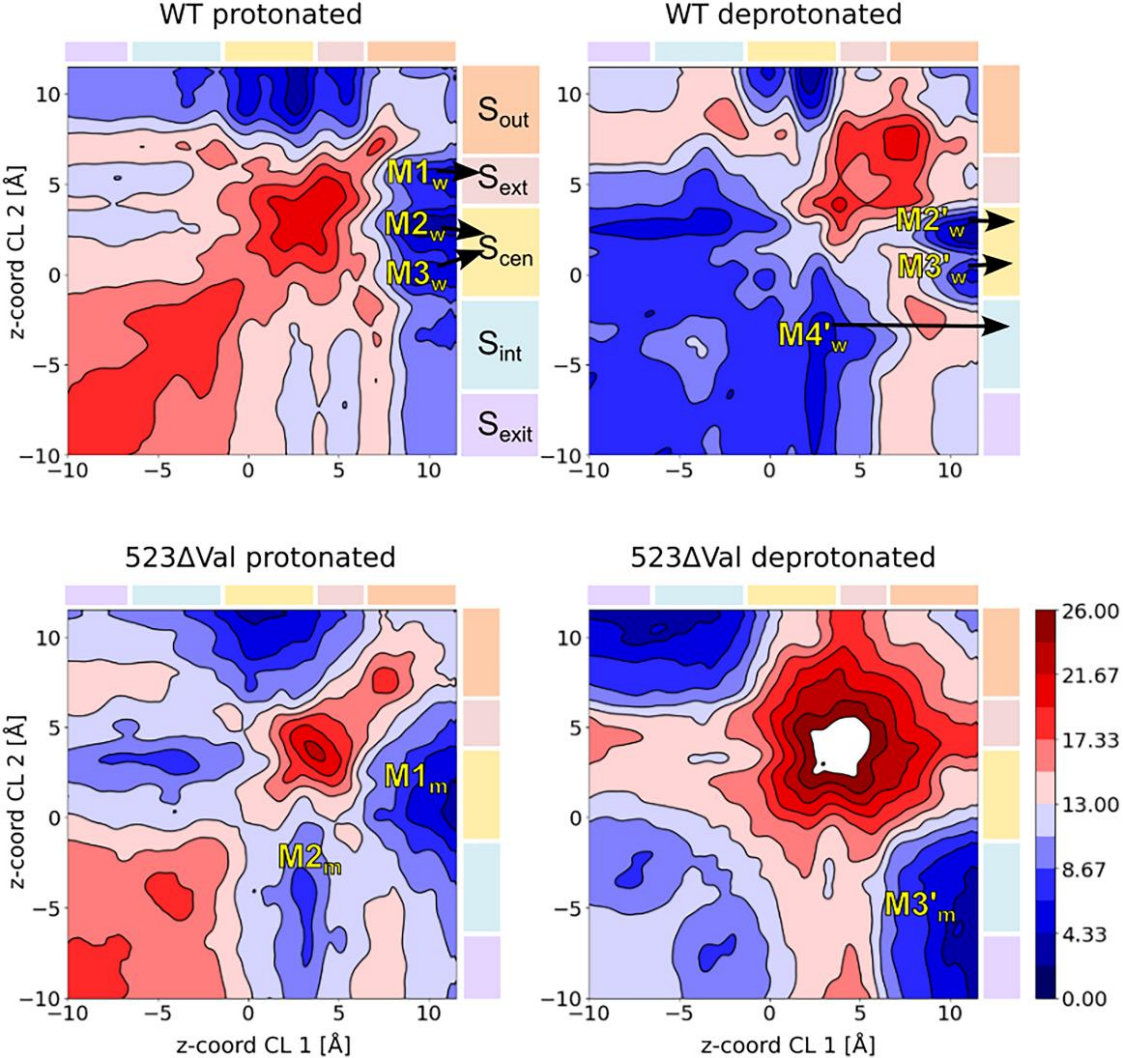
A) Free energy surfaces (FES) obtained from multiple-walker metadynamics simulations of WT and 523ΔVal systems (where W means WT; M the 523ΔVal variant; “protonated” refers to Glu_ext_, while the protonation of Glu_int_ is always opposite). Movement along either the X or Y axis represents the displacement of one Cl- from the extra- (positive values) to the intracellular (negative values) sides; movement along the diagonal means a coordinated movement of the two ions.

Free energy surfaces obtained with MWWTMetaD (see Methods) allowed us to determine the pathway of Cl^−^ migration through the transporter (see Fig. 3A). As shown in the top left panel of Fig. 3A, the first chloride ion can very easily reach a stable binding region defined by three closely placed free energy minima M1_w_ (S_ext_), M2_w_, and M3_w_ (S_cen_), the last of them acting as a kinetic gate. When the first Cl^−^ waits at S_cen_, the second ion can arrive at the extracellular vestibule but is unable to enter further inside the channel due to the presence of the first Cl^−^, which in turn cannot progress to the intracellular side due to a high free-energy barrier. Escape from this stable situation requires a shift in the protonation state of Glu_ext_. According to the WT pK_a_ plot and the constant-pH titration simulations (see Fig. S5), this is indeed what happens: when the bottom chloride reaches S_cen_, and a thermal fluctuation moves the top chloride deeper into S_ext_, the deprotonation of Glu_ext_ becomes more feasible, allowing the residue to release the proton to the extracellular vestibule. A subsequent change in Glu_int_ (Fig. S5) leads to a protonation situation Glu_ext_(deprotonated)-Glu_int_(protonated), which facilitates the migration of the two Cl^−^ as shown in the free energy surface with inverted protonation states (top right panel in Fig. 3A). During deprotonation, the top Cl^−^ ion can enter deeper into the pore, so that the M4′_w_ minimum is reached. In the free energy profiles, this corresponds to a diagonal displacement from M3_w_ (top left) to M4'_w_ (top right), from which both ions can eventually exit the pore through simple diffusion, with large free energy barriers preventing the return of the ions towards the entrance.

**Fig. 4.**
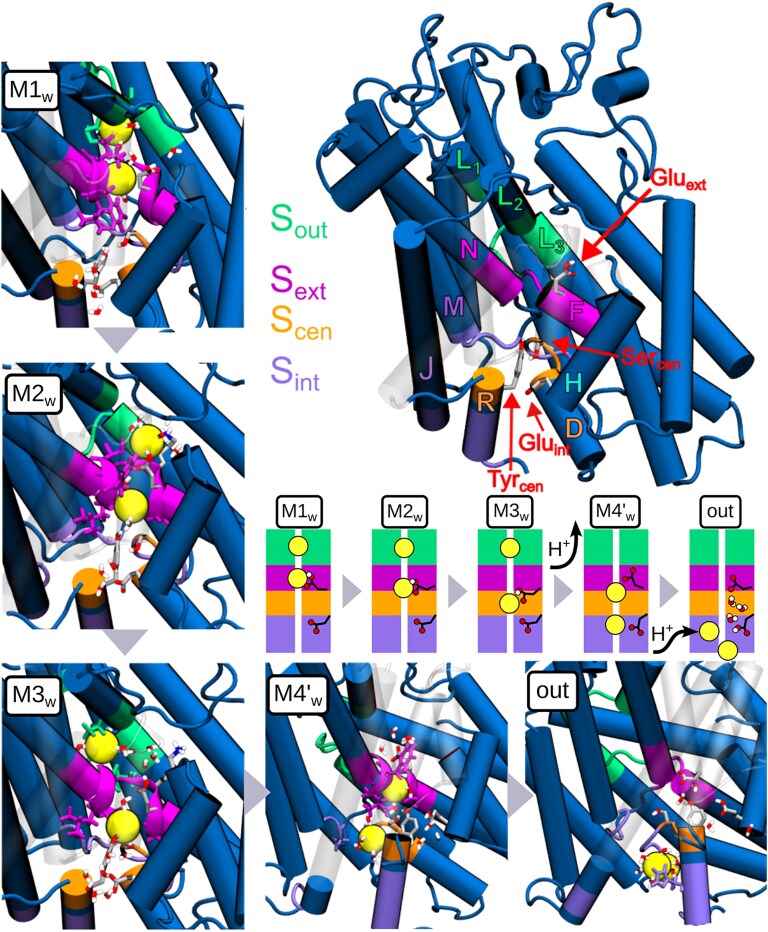
Molecular representation of MWWTMetaD FES minima and anion binding sites of wild-type ClC-5. In the central panel, we represent the location of four identified chloride binding sites in a TM domain. The other panels show a molecular representation of WP (M1_w,_ M2_w_, and M3_w_) and WD (M4_w_ and exit) minima.

A complementary molecular-level explanation of the chlorides' migration is shown in Fig. 4. Initially, extracellular chloride ions are attracted by backbone atoms of loops L1–L2 and E–F, Ser380 and Thr381 of helix L2, and repelled by hydrophobic residues on helix E. After passing the positively charged Lys210, an ion filter known to be a major determinant of anion selectivity (57), the bottom Cl^−^ reaches the S_ext_ cavity, a site enclosed between helices N and F, and protected from extra- and intracellular sides by loops E–F and M–N. At the same time, the other ion can reach the outer side of the channel (S_out_). This defines the M1_w_ state, a minimum in the free energy surface in Fig. 3A. At the second minimum M2_w_, the E–F loop assumes a new conformation, widening the distance between helices F and N. This allows the descent of the Cl^−^ dwelling at S_ext_ to reach the S_cen_ cavity in the neighboring state M3_w_, while the other Cl^−^ remains at S_out_, enlarging the separation between the two chlorides. The S_cen_ binding site is defined by conserved Ser168 (Ser_cen_), Tyr558 (Tyr_cen_), Leu454, Ile170, Phe446, and Leu454 and holds the ion very tightly, hindering the progress of the ion towards the exit. This stable binding site can only be disrupted by the change in the protonation state of Glu_ext_ predicted by pK_a_ maps in Fig. S3, as its side chain reaches the water molecules above the top chloride. Based on our mechanistic observations, we propose that the deprotonation is indeed enabled by the presence of two anions or hydrogen bond acceptors in the binding cavity, where the vertically extended conformation of the Glu_ext_ side chain is stabilized by the “upper” acceptor. This simple observation reconciles the 2:1 stoichiometry of H^+^/Cl^−^ antiport with the ∼1:1 stoichiometry recorded with more elongated anions, such as SCN^−^ or NO_3_^−^ (44).

With the Glu_ext_ deprotonated, the second chloride is forced into the channel, reaching state M4′_w_, followed by a spontaneous diffusion of the two ions through a low stability S_int_ binding site (Fig. 4). In parallel, the now favorable protonation of Glu_int_ prepares the cycle for regeneration. A comparison between the free energy barriers for entry in the top right and top left panels of Fig. 3A shows that the regeneration is crucial to allow the entrance of another pair of chlorides, explaining why the E268A mutant is characterized by a much more drastic ion current loss than E211A (44). In our molecular picture, and consistently with experimental findings (44), the former creates a stalled antiporter by blocking chloride entrance, while the latter would make the “kinetic trap” minima M1_w_, M2_w_, and M3_w_ more shallow, allowing for a relatively fast uncoupled passage of chloride ions.

#### The proton migration

The mechanism outlined above explains the transport of two Cl^−^ from external to intracellular spaces at the expense of a switch in the protonation state of Glu_ext_ and Glu_int_, yielding a net proton transfer from intracellular space to the exterior. Note that when the Cl^−^ transport is completed, the protonation state is: Glu_ext_-protonated and Glu_int_-protonated state, a configuration that would allow for reverse transport from the intracellular space to the cytoplasm (see Fig. 3, WD and WP). This would in turn lead to a futile cycle without a net flux of Cl^−^. However, if the concentration difference of Cl^−^ and/or H^+^ between the two sides of the membrane prevents it, the net flux of ions will continue in the same direction. For the transporter to be regenerated, the proton that was originally transferred from the intracellular space to Glu_int_ must move to Glu_ext_.

**Fig. 5.**
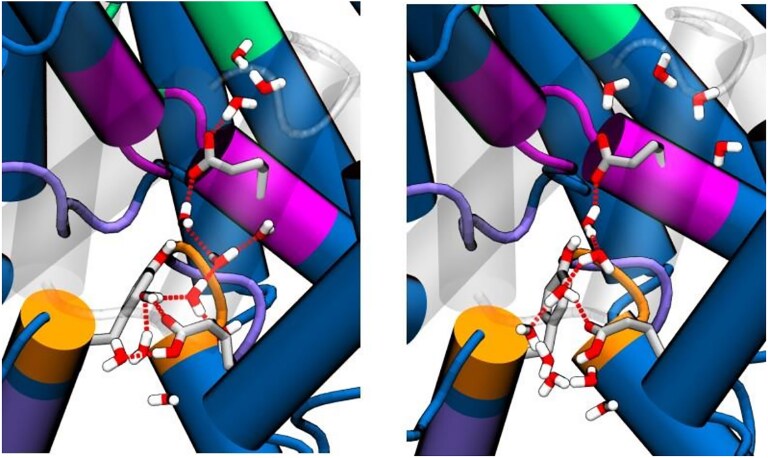
Two examples of a Grotthuss-like water wire connecting Glu_ext_-deprotonated and Glu_int_-protonated that allows for regeneration of the enzyme by recovering the Glu_ext_-protonated and Glu_int_-deprotonated state.

An analysis of the trajectories show that this regeneration can happen through a Grotthus' mechanism, taking advantage of a water spine connecting both glutamic acids when anions are reaching the intracellular vestibule and in equilibrium simulations, i.e. when the respective pK_a_ values favor the exchange (see Fig. 5). Additional QM/MM free energy calculations, reported in Fig. S6, confirm that the concerted proton transfer is thermodynamically viable, with barrier heights almost identical to these reported for model wires of the same length when tunneling is not accounted for (proton transfer rates in the micro- to millisecond range) (58–60). We also note that water wires observed in classical MD simulations are only a convenient proxy, and that wire formation has been found to be significantly enhanced by the presence of a mobile proton (58, 59).

### The impact of the deletion of valine 523

The deletion of Val523 leads to minor changes in the structure: a moderate increase in inter-subunit compaction (Fig. S1A) and a slight displacement of helix P (Figs. 2, S1C, and S2). The impact of such structural changes on the antiporter mechanism is, however, dramatic, consistently with the clinical impact of the mutation. As shown by the MWWTMetaD simulations, the simultaneous transfer of two Cl^−^ as in the WT protein is forbidden (diagonal movement in the bottom left panel of Fig. 3A), but a single Cl^−^ can enter inside the channel so that a state similar to M3_w_ is reached, provided that the external glutamate is in the protonated state. The differences arise when the external glutamate releases a proton to the extracellular vestibule and the internal one takes one from the internal vestibule (i.e. globally Glu_ext_^0^ → Glu_ext_^−^ and Glu_int_^−^ → Glu_int_^0^). In the case of the WT, this change in protonation states of the glutamates leads to a barrier less path for the simultaneous transfer of two Cl^−^ from M3′ to M4′ and the exit (see the favorable free energy values in the bottom left part of the WD plot in Fig. 3A). In turn, the change in protonation states of the two glutamates in 523ΔVal variant leads to a free energy surface where the coordinated transport of two chlorides is forbidden by large free energy barriers, favoring the transfer of a single Cl^−^ ion through the M3′m state and its subsequent diffusion to the intracellular vestibule. This would suggest a one-to-one stoichiometry and an inferior ability to maintain homeostasis. Furthermore, analysis of the pK_a_ plots (Fig. S3) for the different structures collected in the metadynamics simulations shows interesting differences between the WT and deletion variants. As described above, in the WT protein, the entrance of Cl^−^ into the channel is incompatible with Glu_ext_ in the deprotonated form, and the same holds for the 523ΔVal variant (see WT Glu_ext_ and 523ΔVal Glu_ext_). When the two Cl^−^ are reaching the exit, Glu_ext_ becomes very acidic (and accordingly deprotonates) in both WT and deletion variant, even more in the second case (see Fig. S7). Major differences appear however for Glu_int_ between the WT, where it can easily protonate when one Cl^−^ is inside the channel (see blue surfaces in the bottom-left part of the W Glu_int_ plot in Fig. 3B), and the 523ΔVal variant, where very low pK_a_ values hinder protonation when one or even two chlorides are forced to be in the channel (see M Glu_int_ plot in Fig. 3B). In other terms, the change in the protonation state of Glu_int_ required for the two chlorides to reach the exit cavity is much more difficult in the 523ΔVal variant, further compromising the mechanism of dual chloride transport.

**Fig. 6.**
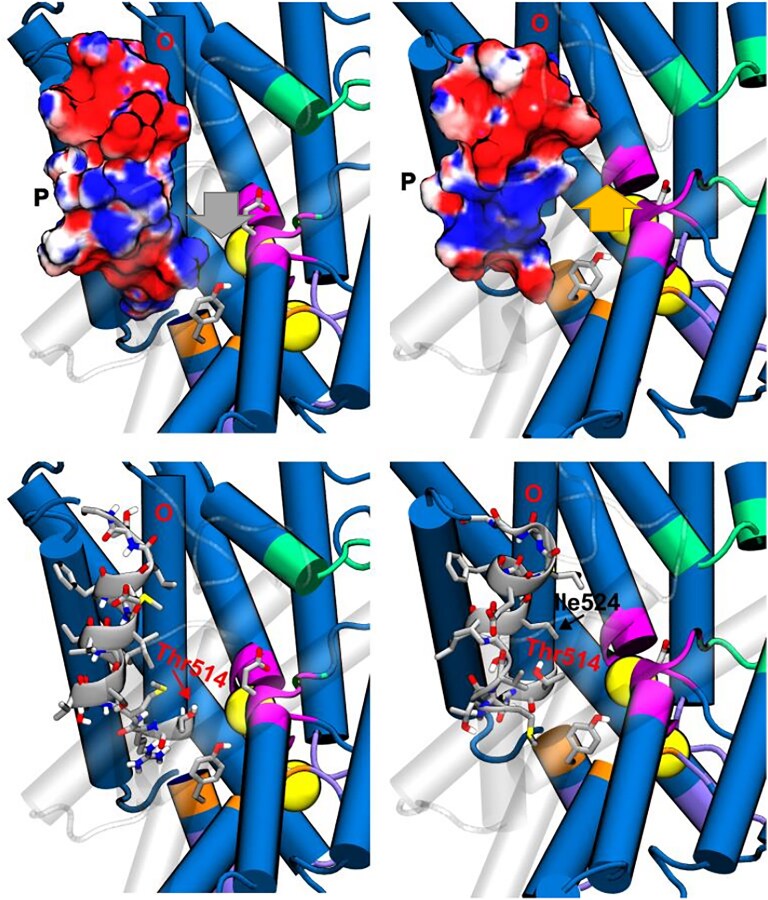
Structural details of the channel in the M4'_w_ region (see Fig. 2), which correspond to a free energy minimum for the WT (left column), but not for the deletion variant (right column). The top row shows electrostatic surfaces, while the bottom row shows side details. Chlorides are shown as yellow spheres.

The structural analysis helps us rationalize the changes induced by an apparently mild deletion that seems to be locally well tolerated (see Figs. S1 and S2). Indeed, the movement of helix P resulting from the deletion leads to (i) altering the local environment of the Glu_int_ (located at the neighboring helix H), which becomes close to Lys174 on helix D, (ii) dehydrating the acidic group, and (iii) stabilizing the anionic state (see Fig. S8). Analysis of the structures around the crucial position (d_Z1_ = 3, d_Z2_=−5) in Fig. 3 shows the impact of the deletion on the tilting of the P helix. This in turn has two major effects: the loss of a close electrostatic interaction stabilizing one of the chloride ions at S_cen_ in the WT but not in the deletion variant (gray arrow in Fig. 6), and the generation of a steric clash for the advance of that chloride in the deletion variant (orange arrow in Fig. 6). Thus, the M4 minimum disappears in the 523ΔVal variant, excluding the possibility for the two ions to reach simultaneously the intracellular vestibule.

**Fig. 7.**
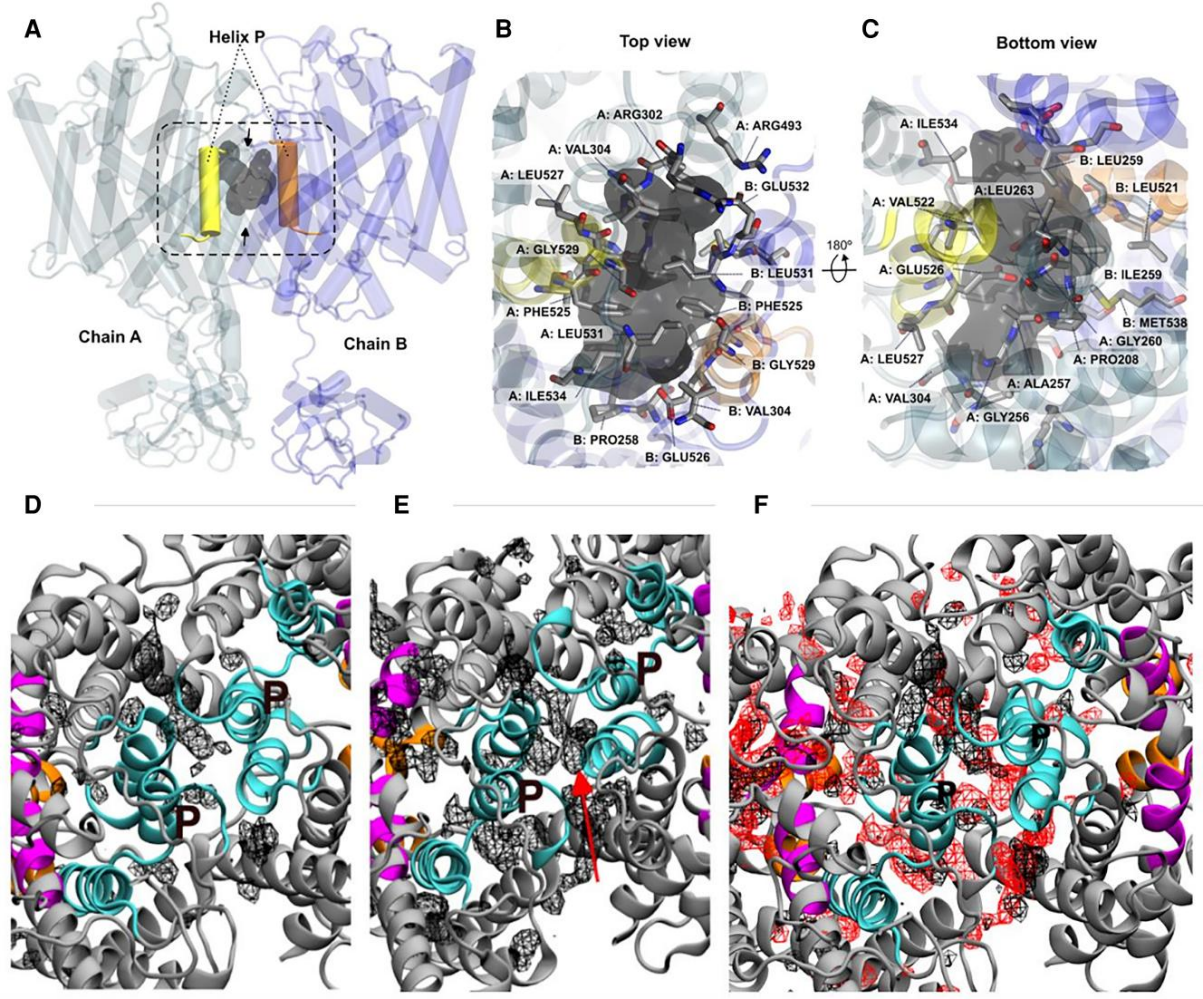
First row: druggable pocket of the representative cluster of the MD for the ValΔ523 mutant variant of ClC-5 homodimer. A, B, C) Calculations were performed using Fpocket on 10 representative structures from the deletion variant (Druggablity score: 0.990. The homodimer is represented in two chains: blue for A (left) and dark blue for B (right). The helix P of chain A and chain B are colored in yellow and orange, respectively. The druggable site candidate is shown with a dark gray surface. A) Global point of view of the druggable site, located between the two helices P. The arrows represent the different points of view described in (B) and (C). B) Zoom in on the druggable site, view from the top, and C) from the bottom. Second row: MDpocket density maps for WT and 523ΔVal variants. Relevant channel helices are in color cyan (helix P labeled), pink, and orange in (D) and (E) the density maps are shown in black wires. D) Density map of the WT protein, using a threshold of 5.7, while in (E) the 523ΔVal variant the threshold is 3.3. The red arrow shows a subpocket only present in the 523ΔVal variant. F) Superimposition of (A) and (D), with WT in black and 523ΔVal in red.

Finally, we explored potential changes in drug-binding properties emerging from the deletion of valine 523. To this end, we ran Fpocket calculations (see Methods) to detect drug-like cavities in WT and 523ΔVal variant. Interestingly, a sizeable cavity with properties that would allow binding of a drug was detected in the vicinity of helix P (see Fig. 7). Fpocket calculations suggest that such a cavity is sensitive to the deletion of valine 523 (Fig. 7A–C), a result that is confirmed by MD calculations using MDpocket (see Methods and Fig. 7D–F), which highlights the presence of variant-specific sub-pockets. Such features could be exploited in the future to develop pharmacological chaperones interacting with the pathological variant to recover the correct alignment of helices P and H, or to more directly modulate the pKa of Glu_int_.

## Conclusions

The transfer of Cl^−^ in antiporters requires a shift in the protonation state of two conserved glutamates located at the two ends of a narrow tunnel controlled by up to four cavities with high residence times for the Cl^−^. According to our free energy calculations, the central site acts as a trap for one Cl^−^, which blocks the entrance of a second one. Escape from this site requires a coordinated change in the protonation state of the external glutamate that interchanges protons with the extracellular vestibule, and the stoichiometry of ion exchange requires the deprotonation to be triggered by the presence of the second chloride. The shift in protonation states leads to a downhill free energy landscape, where two chlorides can migrate almost simultaneously, as well as allows protonating the internal glutamate. This in turn brings the transporter to a final state that can be regenerated by a Grotthuss mechanism involving a short chain of water molecules, with a net flux of one proton from intracellular to extracellular space. In our model, the entire cycle can operate without global conformational changes, relying instead on local rotameric changes of key residues. However, although our aggregate effective sampling time of ca. 0.1 ms is on par with the turnover rate expected for ClC antiporters, one cannot a priori exclude the mechanistic contribution of elusive metastable conformations.

According to our results, the mechanistic disruption caused by the 523ΔVal variant does not rely on global structural rearrangements and is unlikely to produce major changes in stability, distribution, or dimerization properties, but rather induces small local changes in the vicinities of the internal Glutamate and the central site S_cen_. These apparently minor changes (i) modify the effective pKa of Glutamates, making the proton migration very difficult and (ii) change the preferred chloride migration path, making the simultaneous transfer of two chlorides impossible, and hampering a correct homeostasis in the kidney.

## Supplementary Material

pgaf353_Supplementary_Data

## Data Availability

All MWWTMetaD trajectories can be accessed in the MDposit database, acc. number A020P, A020Q, A020R, A020S, available at mdposit.mddbr.eu/#/id/mmb-A020P, mdposit.mddbr.eu/#/id/mmb-A020Q, mdposit.mddbr.eu/#/id/mmb-A020R, and mdposit.mddbr.eu/#/id/mmb-A020S, respectively. Inputs for free energy simulations (PLUMED and QMMM in COLVARS+NAMD) and the starting frames for constant-pH simulations are deposited in https://github.com/mmb-irb/ClC5-data. The script for water wire identification can be accessed at https://gitlab.com/KomBioMol/proton_wire.

## Preprints

This manuscript was posted on a preprint: https://www.biorxiv.org/content/10.1101/2024.11.06.622245v1
