# Supplementary material for: Molecular simulations meet personalized medicine: The mechanism of action of CLC-5 antiporter and the origin of Dent's disease
Source: PNAS Nexus. 2025 Nov 4;4(11):pgaf353. doi: 10.1093/pnasnexus/pgaf353 (PMC12624513; doi:10.1093/pnasnexus/pgaf353)
Supplement: pgaf353_Supplementary_Data [file pgaf353_supplementary_data.pdf]

Supplementary Material

MOLECULAR SIMULATIONS MEET PERSONALIZED MEDICINE. THE MECHANISM OF ACTION OF CLC-5 ANTIPORTER AND THE ORIGIN OF DENT'S DISEASE.

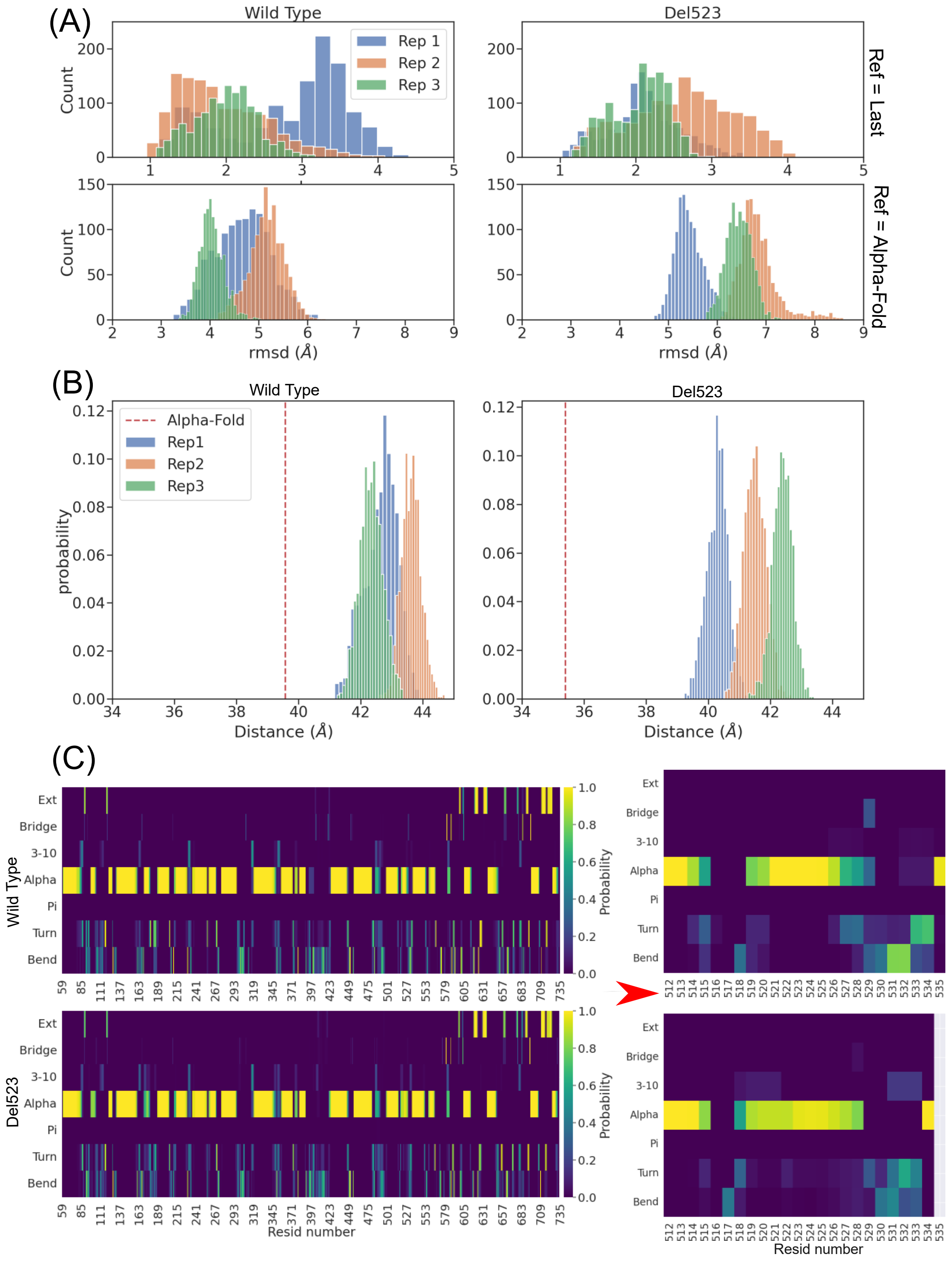

Figure S1: (A) RMSD, (B) Center-of-mass distance and (C) secondary structure analysis carried out over WT and 523ΔVal plain MDs. (A) Backbone-atoms RMSD histograms calculated using the last 500 ns of each plain MD replica (Rep1, Rep2, and Rep3). In the two top panels, the reference is the (WT or 523ΔVal) average structure calculated over the last 100 ns of the corresponding MD replica. In the bottom panels, the reference is the corresponding AlphaFold model structure. (B) Histograms of the distance between the center-of-mass (COM) of the two monomers trans-membrane unit. The COM distance for the corresponding Alpha-Fold model is also reported. (C) Secondary-structure heatmap calculated over each MD replica monomer. In the right panel, a zoom on the α-helix P is shown.

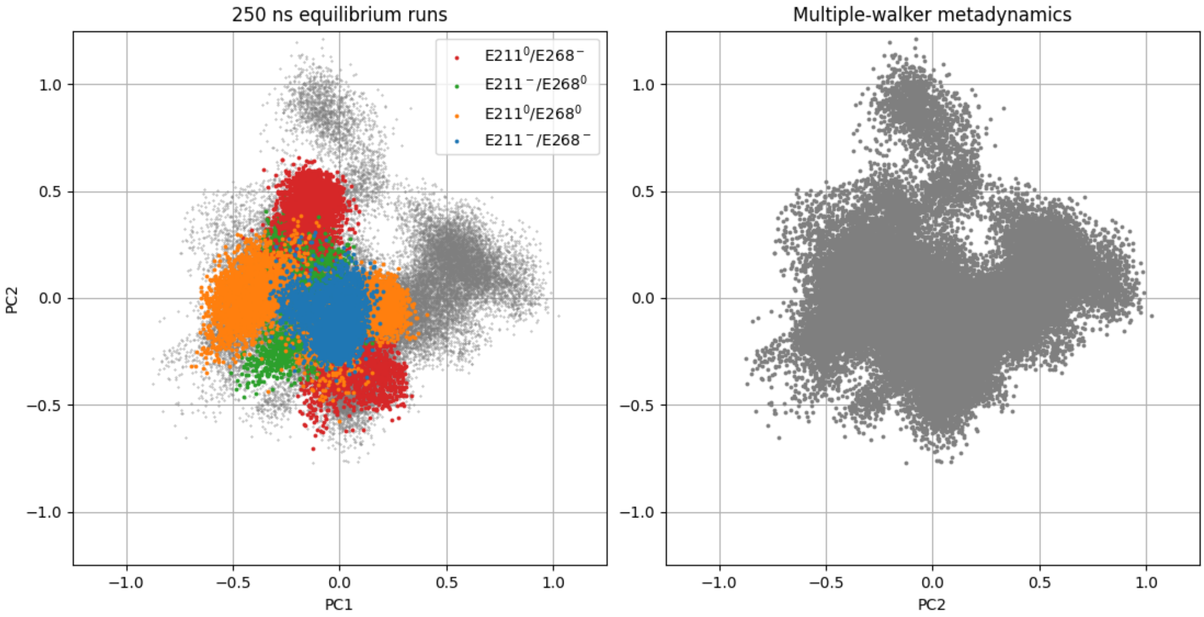

Figure S2: PCA projections obtained from 2x250 ns equilibrium runs initialized with different protonation states of the catalytic glutamates, superimposed on the analogous distribution from the WT multiple-walker metadynamics runs. The analysis was performed on individual chains of the dimer, taking into account alpha carbons within 1 nm of either E211 or E268 (41 residues), i.e., the immediate environment of the central active site.

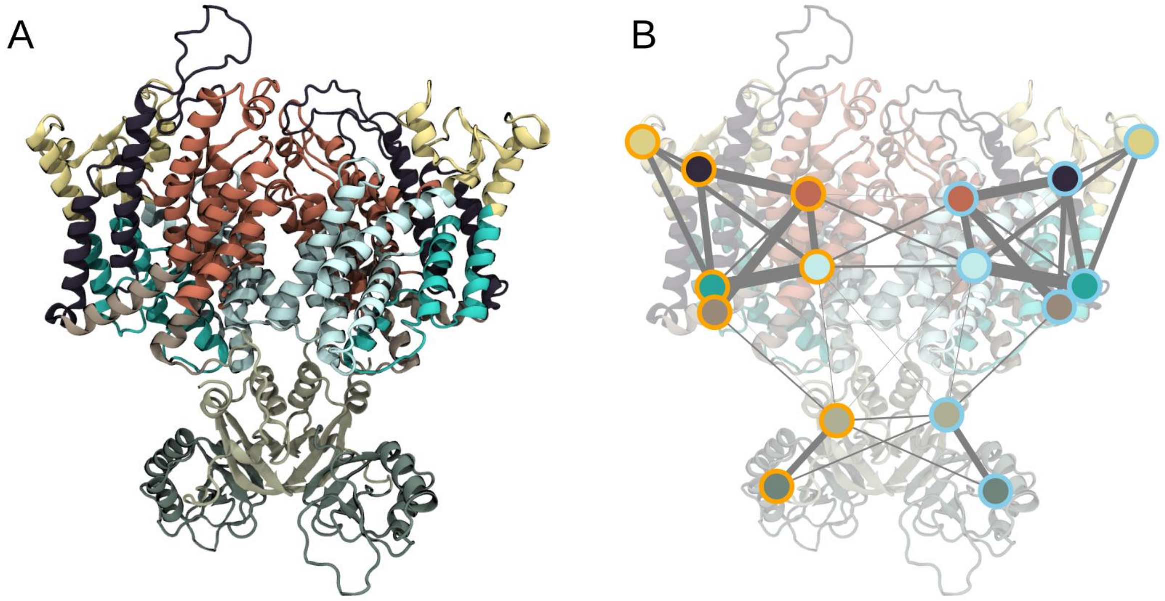

Figure S3: (A) Allosteric communities identified by Dynamic Network Analysis from a representative 250-ns equilibrium ensemble. The assignments of communities are almost perfectly permutation-symmetrical between the two chains without enforcing any symmetry. A default choice of parameters was kept throughout the analysis. (B) Graph representation of inter-community allosteric communication strengths superimposed and color-mapped onto the structure of ClC-5. Here, pairwise interaction strengths were symmetrized between the two chains.

(A)

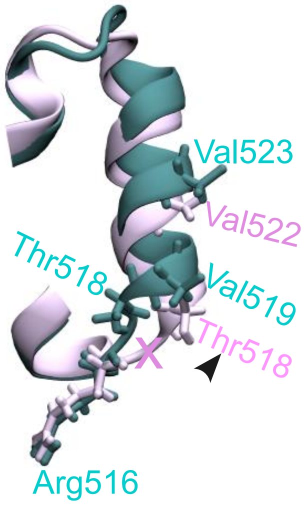

(B)

AA_n	Arg	Met	Thr	Val	Ser	Leu	**Val**	**Val**	Ile	Met	AA_{n+1}
n	516	517	518	519	520	521	522	523	524	525	n
n	516	517		518	519	520	521	522	523	524	n-1
AA_n	Arg	Met		Thr	Val	Ser	Leu	**Val**	Ile	Met	AA_{n+1}

Figure S4: (A) helix-P superimposition of WT and 523ΔVal average MD structures; (B) Alignment of WT and 523ΔVal residues, as observed in plain MD average structure. WT is colored in cyan and 523ΔVal in light pink. Up to Arg515, WT and 523ΔVal perfectly align in both primary and secondary structure. In 523ΔVal, O-P loop Met517 is slightly misplaced from WD, while 523ΔVal Thr518 aligns with WT Val519 on α-helix P. Thus, up to Val522 (WT) and Leu521 (523ΔVal) the secondary structure is conserved, although the amino acid identity is different. Finally, from Val522 and WT Val523 onward, both primary and secondary match again (only the amino acid sequence number is shifted due to the deletion of Val523).

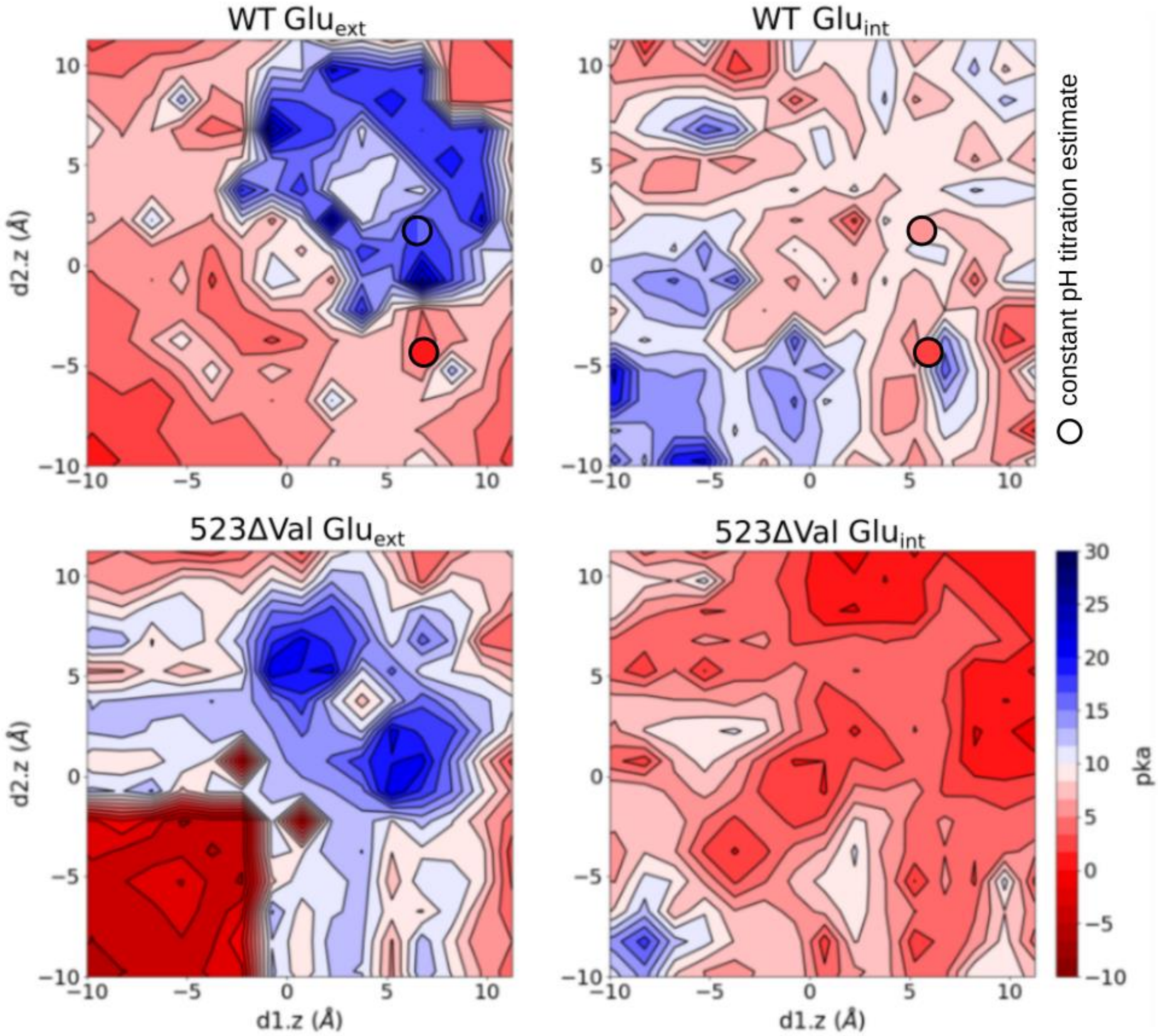

Figure S5: Deprotonation potential maps (used throughout the article as a proxy of pK_a) calculated on the classical CMIP level on a representative set of highest statistical weight frames extracted from MWWTMetaD simulations. The average standard deviation of CMIP-derived pK_a estimates across the 10 chosen frames was 1.7 pH unit for Glu$_{int}$ and 3.0 pH units for Glu$_{ext}$. Circles denote pK_a estimates derived from titrations employing constant-pH molecular dynamics (>14 and 1.1 for Glu$_{ext}$; 5.8 and 3.0 for Glu$_{int}$).

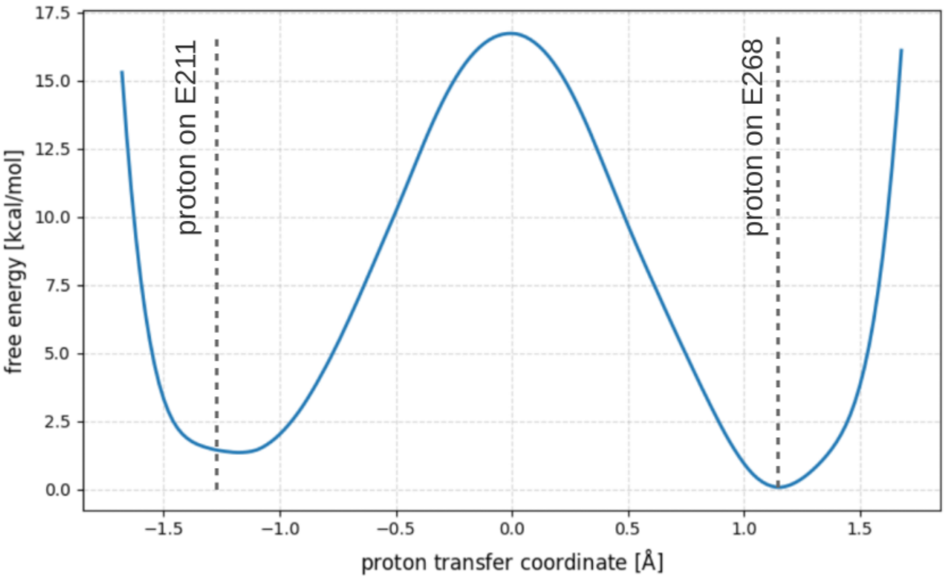

Figure S6: A free energy profile for the concerted proton transfer in a proton wire (see Fig. 5) connecting Glu_{int} and Glu_{ext}. Calculations were performed at the semi-empirical PM6-D3H4X level using the NAMD-MOPAC interface, and the final plot is an average of 9 intermediate free energy profiles. The free energy barrier of 16.5 kcal/mol is comparable to those reported in the literature by Kaila and Hummer (12.7 kcal/mol) and Li and Voth (15-18 kcal/mol), albeit somewhat larger than reported for ClC-ec1 by Lee, Swanson and Voth in the absence of the chloride (10.2-10.9 kcal/mol).

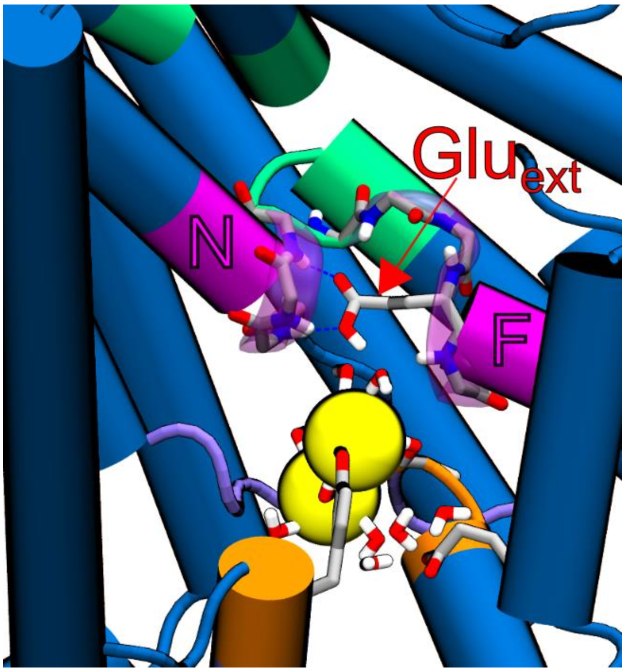

Figure S7: Interactions of deprotonated Glu_{ext} with the NH backbone of helix N, F (including the Glu_{ext} NH backbone itself), and residues on E-F loop. This snapshot was extracted from multiple-walker metadynamics simulations, system MP.

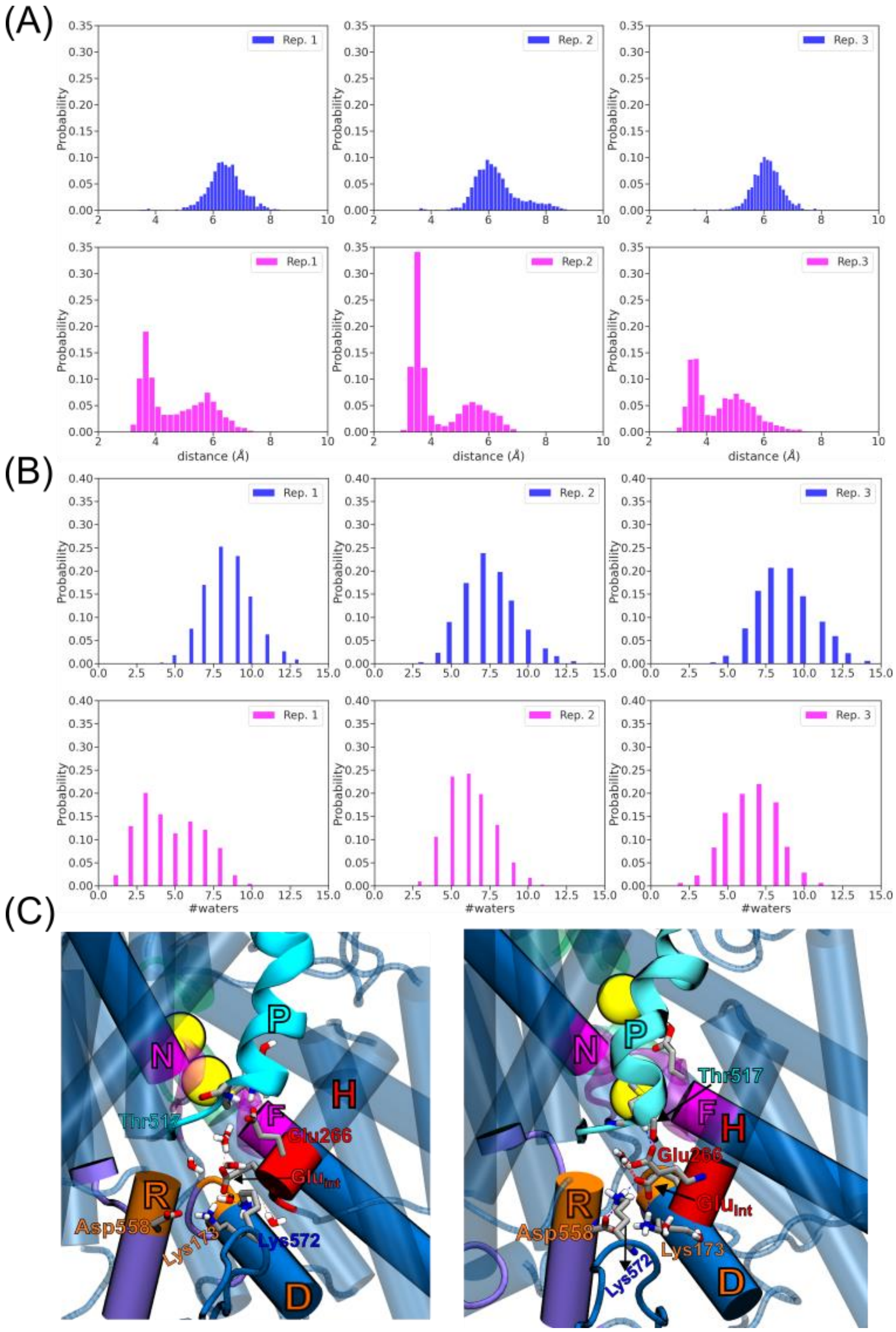

Figure S8: (A) Distance between Lys172(NH3) and Glu_{int}(OE1, OE2) in 3 replicas of WD (top) and MD (bottom) plain Molecular Dynamics simulations. Note that for each replica, results from chain A and chain B have been plotted together. (B) Number of water molecules (#waters) around Glu_{int}(OE1, OE2) in 3 replicas of WD (top) and MD (bottom) plain Molecular Dynamics simulations. Note that for each replica, results from chain A and chain B have been plotted together. (C) Representation of interactions between Thr517 (helix P) and Glu266 (helix H), as well as between Lys173 (helix D) or Lys572 (TM-CBS loop) and Glu_{int}. Frames have been extracted from M1w and M1m minima.

Table S1: Mean RMSDs between the transmembrane part of the starting structure generated by AlphaFold-2 and published structures of proteins belonging to the ClC family. The RMSD was

calculated using the STAMP alignment tool as implemented in VMD, and averaged over all residues that were included in the alignment. For each dimeric structure, only chain A was considered in the calculations. Transporters are highlighted in light blue, and channels in light yellow.

protein	human ClC-3	human ClC-2	Human ClC-6	human ClC-7	algal ClC	human ClC-1	E.coli ClC
AF2 vs	9DO0	7XF5	8JPO	7JM7	3ORG	6QV6	1OTS
RMSD [Å]	0.699	1.544	1.565	1.628	1.653	1.782	2.060

Supplementary Methods

Definition of the collective variable for MWWTMetaD. The 2-dimensional collective variable was defined in Plumed[1] as the Z-distance between each of the selected chloride ions and the center of mass of the residues forming the central cavity, S_{cen}, chosen as alpha and carbonyl carbon atoms of residues G169, I170, P171, K210, E211, G212, L454, F455, I456, P457, V514, T515, I557 and T558. Due to permutational symmetry, this choice allowed us to augment the obtained data by swapping the two dimensions. In addition, the movement of the ions was restrained in the XY plane to within 6 Å from the respective center of mass of the S_{cen} residues to avoid uncontrolled ion escape close to the top and bottom of the channel. Ions were allowed to sample the Z-distance interval -20 Å < z < 11.5 Å, with semi-harmonic walls with a force constant of 2000 kJ/Å²mol set to all such restraints. In well-tempered metadynamics, Gaussian kernels were deposited every 1000 MD steps with an initial height of 1 kJ/mol, a width parameter of 0.3 Å, and a bias factor of 10. The reweighting factor[2] was calculated on the fly to enable subsequent reconstruction of the free energy surfaces and selection of high-probability frames for the pKa calculation.

Preparation and equilibration of membrane systems. CHARMM-GUI Membrane Builder[3] was used to set up the membrane-embedded system for Gromacs. The standard multi-step protocol, including energy minimization and a series of equilibrations with gradually reduced restraints, was performed prior to long equilibration; the latter was extended to 1 microsecond for each system in order to identify any deletion-induced structural changes not captured by AlphaFold-Multimer. For each of the two systems (WT and 523ΔVal), 4 frames were selected to ensure maximum variability of initial chloride ion positions, and indices of the two chosen ions were swapped to create symmetric starting points in the 2-dimensional space. For the free energy calculations, excess ions were removed to adjust ion concentration to the physiological value of 0.15M; the final systems contained ca. 57500 TIP3P water molecules, 160 K^+ and 156 Na^+ ions, as well as 387 POPC residues.

Details of the CMIP approach and pKa calculation. CMIP[4] is a method for calculating a classical molecular interaction potential, defined as a sum of the van der Waals and Coulombic potentials around a macromolecule. In the context of pKa calculations, it was used to compute the relative energetic cost of removing a proton from a protonated residue, and this cost can be translated into the offset from the pKa of an isolated amino acid through the familiar expression:

$$\Delta pK_a = \frac{\Delta E_{cmip}}{2.303RT}$$

The calculation was performed using a suite of in-house Perl scripts that were previously applied to calculate pKa in [5].

Detection of druggable pockets using MDpocket. MDpocket[6] is an algorithm based on Fpocket[7] that accounts for the plasticity of the pockets along a molecular dynamic simulation. Unbiased simulations were pre-aligned on the transmembrane region of the A monomer. The last 500 ns of 3 replicas for each variant were used to obtain the map of normalized densities and frequencies of alpha-spheres by following the standard pocket characterization protocol of MDpocket (https://github.com/Discngine/fpocket). Results were inspected using the Visual Molecular Dynamics viewer[8] and cavities were identified by slowly increasing the isovalues from the density map until the pocket located between helices P was formed and differentiated. Therefore, the isovalue was set to 5.7 for the WT and 3.3 for the Δ523 variant (Figure 7DEF).

BIBLIOGRAPHY

1. Tribello, G. A., Bonomi, M., Branduardi, D., Camilloni, C. & Bussi, G. PLUMED 2: New feathers for an old bird. *Comput Phys Commun* **185**, 604–613 (2014).
2. Tiwary, P. & Parrinello, M. A time-independent free energy estimator for metadynamics. *Journal of Physical Chemistry B* **119**, 736–742 (2015).
3. Wu, E. L. *et al.* CHARMM-GUI *Membrane Builder* toward realistic biological membrane simulations. *J Comput Chem* **35**, 1997–2004 (2014).
4. Gelpí, J. L. *et al.* Classical molecular interaction potentials: Improved setup procedure in molecular dynamics simulations of proteins. *Proteins: Structure, Function, and Bioinformatics* **45**, 428–437 (2001).
5. Serrano-Chacón, I. *et al.* pH-Dependent Capping Interactions Induce Large-Scale Structural Transitions in i-Motifs. *J Am Chem Soc* **145**, 3696–3705 (2023).
6. Schmidtke, P., Bidon-Chanal, A., Luque, F. J. & Barril, X. MDpocket: open-source cavity detection and characterization on molecular dynamics trajectories. *Bioinformatics* **27**, 3276–3285 (2011).
7. Le Guilloux, V., Schmidtke, P. & Tuffery, P. Fpocket: An open source platform for ligand pocket detection. *BMC Bioinformatics* **10**, 168 (2009).
8. Humphrey, W., Dalke, A. & Schulten, K. VMD: Visual molecular dynamics. *J Mol Graph* **14**, 33–38 (1996).
